# Identification of Molecular Markers of Delayed Graft Function Based on the Regulation of Biological Ageing

**DOI:** 10.1371/journal.pone.0146378

**Published:** 2016-01-06

**Authors:** Dagmara McGuinness, Johannes Leierer, Olivier Shapter, Suhaib Mohammed, Marc Gingell-Littlejohn, David B. Kingsmore, Ann-Margaret Little, Julia Kerschbaum, Stefan Schneeberger, Manuel Maglione, Silvio Nadalin, Sylvia Wagner, Alfred Königsrainer, Emma Aitken, Henry Whalen, Marc Clancy, Alex McConnachie, Christian Koppelstaetter, Karen S. Stevenson, Paul G. Shiels

**Affiliations:** 1 University of Glasgow, College of Medical, Veterinary & Life Sciences, Wolfson Wohl Translational Research Centre, Institute of Cancer Sciences, Garscube Estate, Switchback Road, Glasgow, G61 1QH, Scotland; 2 NHS Greater Glasgow and Clyde, Renal Transplant Unit, Ward 4c, South Glasgow University Hospital, Glasgow, G51 4TF, Scotland; 3 NHS Greater Glasgow and Clyde, Histocompatibility and Immunogenetics, Laboratory Medicine Building, Gartnavel General Hospital, Glasgow, G12 0XL, Scotland; 4 Center of Operative Medicine, Department of Visceral, Transplant and Thoracic Surgery, Innsbruck Medical University, A-6020 Innsbruck, Austria; 5 Universitätsklinikum Tübingen, Universitätsklinik für Allgemeine, Viszeral- und Transplantationschirurgie, Transplantationszentrum, D-72076 Tübingen, Germany; 6 Universitätsklinikum Tübingen, Klinik für AllgemeineViszeral und Transplantationschirurgie, Chirurgische Studienzentale, D-72076 Tübingen, Germany; 7 Universitätsklinikum Tübingen, Universitätsklinik für Allgemeine, Viszeralund Transplantationschirurgie, CRONA, D-72076 Tübingen, Germany; 8 Robertson Centre for Biostatistics, University of Glasgow, Glasgow, Scotland; University of Torino, ITALY

## Abstract

**Introduction:**

Delayed graft function is a prevalent clinical problem in renal transplantation for which there is no objective system to predict occurrence in advance. It can result in a significant increase in the necessity for hospitalisation post-transplant and is a significant risk factor for other post-transplant complications.

**Methodology:**

The importance of microRNAs (miRNAs), a specific subclass of small RNA, have been clearly demonstrated to influence many pathways in health and disease. To investigate the influence of miRNAs on renal allograft performance post-transplant, the expression of a panel of miRNAs in pre-transplant renal biopsies was measured using qPCR. Expression was then related to clinical parameters and outcomes in two independent renal transplant cohorts.

**Results:**

Here we demonstrate, in two independent cohorts of pre-implantation human renal allograft biopsies, that a novel pre-transplant renal performance scoring system (GRPSS), can determine the occurrence of DGF with a high sensitivity (>90%) and specificity (>60%) for donor allografts pre-transplant, using just three senescence associated microRNAs combined with donor age and type of organ donation.

**Conclusion:**

These results demonstrate a relationship between pre-transplant microRNA expression levels, cellular biological ageing pathways and clinical outcomes for renal transplantation. They provide for a simple, rapid quantitative molecular pre-transplant assay to determine post-transplant allograft function and scope for future intervention. Furthermore, these results demonstrate the involvement of senescence pathways in ischaemic injury during the organ transplantation process and an indication of accelerated bio-ageing as a consequence of both warm and cold ischaemia.

## Introduction

Increasing demand for renal transplantation has resulted in the use of kidneys which previously would have been considered marginal and this combined with an increase in Donation after Circulatory death (DCD) has increased the prevalence of renal allografts at risk of Delayed graft function (DGF). DGF is most commonly defined as the need for dialysis within one week post-transplant [1,2]. DGF is a consequence of Acute Kidney Injury (AKI) related to peri-transplant processes and results in prolonged post-operative hospitalisation. Although previous work has reported no deleterious impact of DGF on long term survival, it remains to be seen if this is maintained in the context of a changing donor population. Consequently, this important prognostic factor for allograft outcome is likely to remain a significant clinical challenge for the foreseeable future, as the use of Extended Criteria Donors (ECD) and DCD kidneys to meet the shortfall in available kidneys on a growing transplant waiting list, is associated with an increased incidence of DGF [3,4]. Furthermore, although our understanding of the clinical risk factors has improved this has not translated into the development of therapies to attenuate or treat DGF.

Until recently, donor chronological age has been the most widely accepted and utilised criterion in assessment of an organ for transplantation. We, and others, have demonstrated that allograft biological age, determined by the expression level of CDKN2A pre-transplant, is a superior prognostic and predictive marker to donor age in this context [5,6,7]. Notably, however, expression of this marker, has already shown an association with DGF in one study [5], opening an avenue to an improved molecular characterisation for this problem and thus better risk benefit analysis and better informed patient management.

The CDKN2 locus is complex and subject to a range of spatio-temporal and developmental epigenetic regulation, which integrates expression of its gene products to a range of stress and damage related biochemical pathways (e.g. mTOR, P13K, TGF-β). Recent studies have implicated epigenetic regulation by microRNAs as critical regulators of the CDKN2 locus, renal development as well as physiological processes [8,9]. Indeed, they have been used to provide a valuable post-transplant signature of renal function and allograft rejection.

MicroRNAs are small (18–25 nucleotides), non-coding, single-stranded RNA molecules that are involved in the regulation of a variety of biological processes, including embryogenesis, differentiation, and senescence [10,11,12]. MicroRNAs are also critical for mRNA stability and are involved in the regulation of gene silencing [13]. This has led us to hypothesis that miRNAs associated with such pathways may be potential biomarkers for DGF. Two miRNAs, hsa-mir-217 and hsa-mir-125b, involved in cellular stress and damages responses are of particular interest in this context, as they help regulate biological ageing processes common across taxa and as mediators of CDKN2 loci transcript expression.

In this study, we have sought to determine in a retrospective analysis of pre-transplant allograft biopsies, if these miRNAs associate with DGF, or where interrelated with pre-transplant parameters that might influence outcome, or post-transplant performance (e.g. cold ischaemia time, type of organ donation, ECD) that might impact upon DGF.

We have hypothesised that such a signature may identify molecular characteristics that offer insight into the clinical phenotype of delayed graft function and potential novel therapeutic targets.

## Materials and Methods

### Renal allograft recipients and Biopsy specimens

Cortical needle biopsies of renal allografts were obtained pre-transplant (N = 94, placed in RNA®*later*(Ambion, USA) and stored at -80°C for further analysis. Donor and recipient characteristics are shown in Table 1. This study as well as consent procedure was approved by the Regional Ethics Committee of North Glasgow NHS Trust (GN10SU334, 10/S0704/42). Donors from the national pool donated their organs for transplantation. The recipient of the organ provided pre-operative written informed consent and records are stored at South Glasgow University Hospital.

**Table 1:** Patient clinical and experimental characteristic. Continuous variables are expressed as mean with standard deviation, whereas categorical variables are expressed as proportions.

Variable	Mean (Min-Max)/ Proportion	Standard deviation (if applicable)
**DONOR**		
**Donor gender (males/females)**	52/42	
**Donor age (years)**	46.6 (11–78)	17.8
**Donor serum creatinine at retrieval (μmol/L)**	92.2 (23–786)	84.4
**Ethnicity (Caucasian: Asian)**	93:1	
**Donor type**		
DBD/DCD	67/27	
ECD	32	
DBD-ECD	24	
DCD-ECD	8	
**Cause of Death**		
Intracranial Haemorrhage	43	
Hypoxic Brain Injury	26	
Trauma	9	
Tumour	5	
Meningitis	4	
Intracranial Thrombus	6	
Respiratory Failure	1	
**Donor hypotension (N)**	62	
**Donor hypertension (N)**	54	
**RECIPIENT**		
**Recipient gender (males/females)**	59/35	
**Recipient age (years)**	50.7 (20–75)	12.5
**Aetiology of Renal Failure**		
IgA Nephropathy	14	
Glomerulonephritis	14	
APKD	19	
Reflux /Obstructive Uropathy	9	
Hypertension	4	
Diabetes Mellitus	6	
Unknown	12	
Chronic pyelonephritis	4	
HSP	1	
Alport’s Syndrome	1	
Reno-vascular	2	
Drug related	2	
Stone disease	2	
SLE	2	
HUS	1	
FSGS	1	
**Previous Transplant**		
0	77	
1	15	
2	2	
**HLA Mismatch**		
HLA- A (0/1/2)	29/38/27	
HLA- B (0/1/2)	33/50/11	
HLA- DR (0/1/2)	67/24/3	
**Cold ischaemic time (hours)**	13.7 (6–23)	3.9
**Immunosuppression**		
**Induction**: Basiliximab/ Campath /ATG	80/7/5	
**Maintenance**: Tacrolimus /Sirol/Cyc	92/1/1	
Prednisolone	88	
Mycophenolatemofetil	87	
**BPAR**	16	
**DGF**	27	
**Serum creatinine level at 6 months (μmol/L)**	125.5 (55–318)	53.2
**MDRD4 at 6 months (ml/min/1.73m**^**2**^**)**	59.4 (17.6–115.3)	23.1
**Serum creatinine level at 1 year (μmol/L)**	128.8 (55–377)	57.3
**MDRD4 at 12 months (ml/min/1.73m**^**2**^**)**	59 (16.2–120)	22.2

Samples were anonymised and subsequently analysed. The validation cohort had a separate ethical approval granted (Innsbruck Medical University and Universitätsklinikum Tübingen) and full written consent was collected for each subject (Table A in S1 Data.). None of the transplant donors were from a vulnerable population and all donors or next of kin provided written informed consent that was freely given.

Total RNA from kidney biopsies was extracted using TRI^®^ Reagent according to the manufacturer’s instructions (Invitrogen, UK) and stored at -80°C for further analysis. Quantitative and qualitative analysis of isolated RNA was performed for each sample; this included spectral analysis (A_260/280 nm_), non-denaturing agarose electrophoresis and expression profile of 18S rRNA. RNA extraction for validation cohort can be found in S1 Data.

### MicroRNA expression and data analysis

MicroRNA profiles were determined in pre-transplant renal biopsies using micro-fluidic cards comprising of 754 independent microRNAs (TaqMan® Array Human MicroRNA A+B Cards Set v3.0, Applied Biosystems). 10 renal biopsies were used for profiling studies (5 good performing allografts and 5 poor performing allografts).

1 μg of total RNA for each card in the set was reverse transcribed using Megaplex™ Primer Pools, Human Pools Set v3.0 and TaqMan® MicroRNA Reverse Transcription Kit according to the manufacturer’s instructions (Applied Biosystems). QPCR was carried out on 7900HT Real-Time PCR system (Applied Biosystems) using thermal profiles recommended by the manufacturer. Amplification profiles for each sample and target were analysed individually and profiles with Ct above 35, bad passive reference signal, noise spike and high noise were removed from further analyses. Data analysis was performed using RQ Manager 1.2 and RealTime®StatMiner (Integromics). Data were normalised against the following endogenous controls: RNU44, RNU48 and MammU6 or U6snoRNA depending on the card used. The comparative threshold cycle method (ΔΔCT) was used to quantify relative gene expression, and the obtained quantification was transformed to exponential value 2^-ΔΔCT^ [14]. MicroRNA expression profile generation for validation cohort is described in S1 Data.

### QPCR

For each individual RT reaction 10 ng of total RNA from each sample was used and reverse transcription was performed using *TaqMan*^®^MicroRNA Reverse Transcription Kit (Life Technologies Inc., UK). RT product was further amplified using individual microRNA®*Taqman* assays. hsa-miR-217 (002337) and hsa-miR-125b (000449) expression was normalised against the following endogenous controls: RNU44 (001094), RNU48 (001006) and U6snoRNA (001973) for each sample.

For gene expression analysis, 1 μg of total RNA was reverse transcribed using SuperScript®II Reverse Transcriptase (Life Technologies Inc., UK) and then qPCR was performed. The gene expression was analysed using TaqMan^®^gene expression assays for CDKN2A (Hs00923894_m1), ANRIL (Hs04406279) and normalised against HPRT1 (Hs02800695_m1). The comparative threshold cycle method (ΔΔ CT) was used to quantify relative gene expression, and the obtained quantification was transformed to exponential value 2^- ΔΔ CT^ [14]. RNA extracted from the kidney of a 41 year old healthy donor was used as a calibrator for all experiments.

### Statistics

Statistical analysis of microRNA expression was performed using the SPSS statistical package (v17). Spearman correlations and appropriate parametric or non-parametric tests were used to compare variables.

Logistic regression analyses were performed to demonstrate any association between differential microRNA expression in relation to DGF as well as biochemical and clinical parameters. This was accomplished using a backward stepwise approach, where all variables were added to the model, and removed based on significance of input to the model. p<0.05 was considered significant.

## Results

94 pre-perfusion renal biopsies collected upon arrival for transplantation from deceased donors were included in this study. Patient characteristics are described in Table 1. All kidneys in this cohort were subsequently transplanted. Mean donor age was 46.6 years and 55% were from male donors. 28.7% were donors after circulatory death and 34% of all donors were extended criteria donors. 4 kidneys had AKI at retrieval with serum Creatinine levels of > 200 μmol/L. However, none of these kidneys displayed delayed graft function post-transplantation. The overall DGF rate in this cohort is 28.7%. The mean cold ischaemic time is 13 hours which compares well with national averages and limits one of the known risk factors for DGF in this cohort.

MicroRNA profiles were determined in pre-transplant biopsies using micro-fluidic cards comprising 754 independent microRNAs (TaqMan^®^ Array Human MicroRNA A+B Cards Set v3.0, Applied Biosystems). For initial profiling, 10 pre-transplant renal allograft biopsies were used; of these 5 were good performers within 2 years of transplant while the other 5 presented poor function within the same time frame. Differential expression between good and poor performers (minimum of two-fold difference) was observed for hsa-miR-505, hsa-miR-34a, hsa-miR-1275, hsa-miR-125a-5p, hsa-miR-449a, hsa-miR-155, hsa-miR-101#, hsa-miR-375, hsa-miR-96, hsa-miR-217 and hsa-miR-125b. These microRNAs were further analysed in the context of other clinical parameters including DGF, serum Creatinine, MRDR4 at 6 and 12 months post-transplant and type of organ donation.

### Association analysis

**Fig 1:**
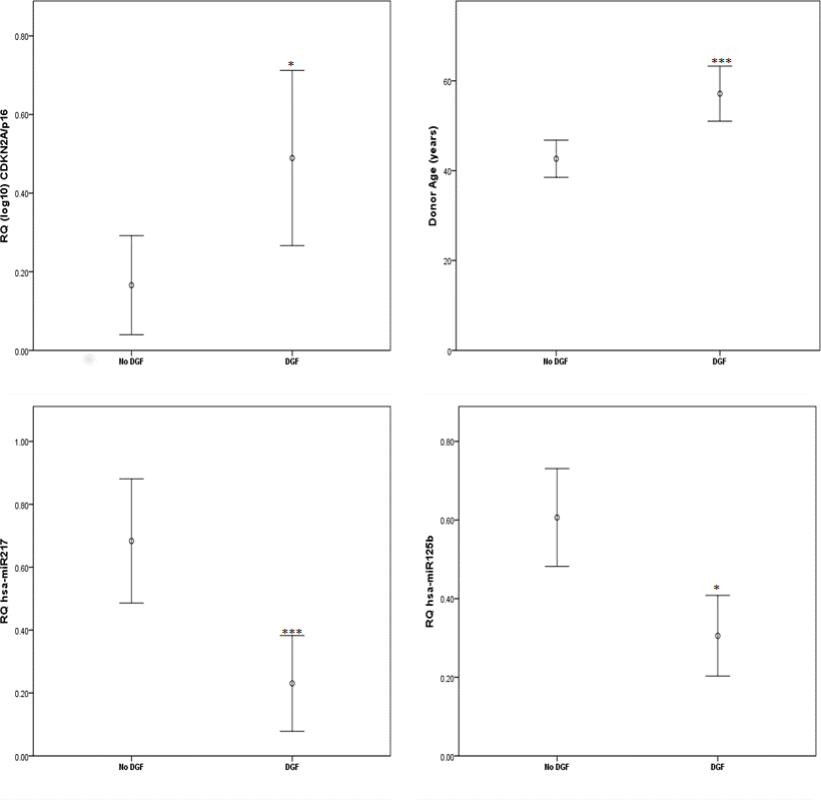
Delayed graft function is related to donor age, CDKN2A/p16 and the expression of hsa-miR-217 and hsa-miR-125b. Gene and MicroRNA expression was assayed using individual TaqMan®assays and normalised against the following endogenous controls: RNU44, RNU48 and U6snoRNA (for microRNA) and HPRT1 for CDKN2A. Data presented as mean with 95%CI.

The incidence of DGF was associated with higher donor age (p<0.001), increased CDKN2a/p16 expression (p = 0.01) and decreased expression of hsa-miR-217 (p<0.0001) and hsa-miR-125b (p = 0.035) in allografts showing DGF post-transplant compared to these without the occurrence of DGF (Fig 1). Interestingly, a negative correlation between hsa-miR-217 and CDKN2A/p16 expression was also noted (cc = -0.392, p = 0.001). Further analysis of the CDKN2 locus revealed that hsa-miR-217 is positively correlated with ANRIL (CDKN2B-AS1, cc = 0.289, p = 0.029).

Lower hsa-miR-125b (p = 0.024) was observed in donors after cardiac death as compared to donors after brain death, this microRNA was also positively correlated to cold ischaemia time (cc = 0.227, p = 0.028)

**Fig 2:**
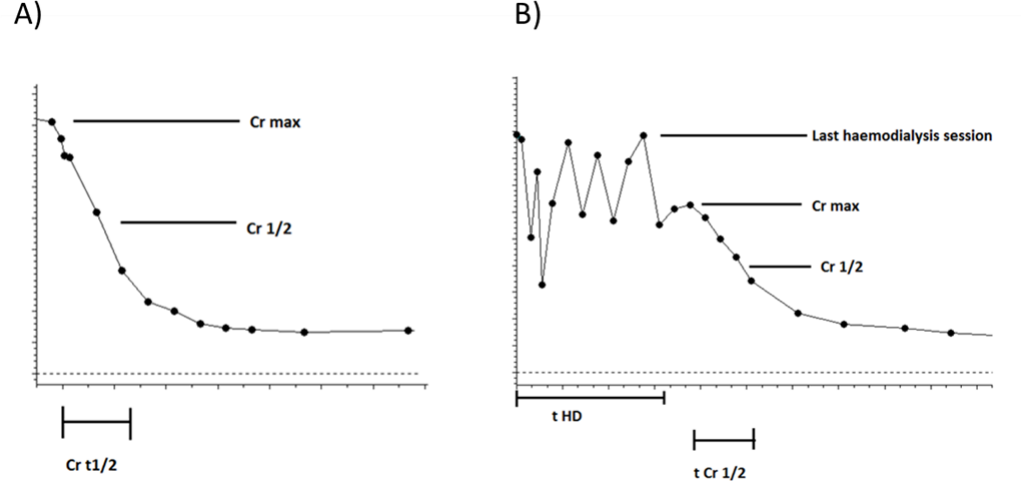
Cr t1/2 calculation and post-transplant organ recovery. A) Cr t1/2 for immediately functioning grafts, B) Cr t1/2 for DGF requiring haemodialysis.

We have also analysed the expression of differentially expressed transcripts and microRNAs in the context of recovery from transplant related injury measured as the time for serum creatinine levels to half (Cr t^1^/_2_)[15]. This is defined as the time taken for the serum creatinine level to drop to half of its peak post-operative value. For immediate functioning grafts this will be the highest value seen post-transplant. For those with DGF, the peak value is taken as the highest creatinine value observed following the last haemodialysis session (Fig 2). Cr t1/2 was significantly correlated with the expression of hsa-miR-125b (cc = -0.287, p = 0.006) and allograft function measured as MDRD4 at 3 months (cc = -0.4010, p<0.0001) and 6 months post-transplant (cc = -0.285, p = 0.006; Table 2). The expression of hsa-miR-217 was positively correlated with MDRD4 at 12 months (cc = 0.300, p = 0.007; Table 2)

**Table 2:** Correlation matrix of clinical outcome parameters and miRNA expression.

Spearman's rho		Donor age	CIT(h)	Cr t 1/2	MDRD4_3m	MDRD4_6m	MDRD4_12m
**hsa-miR-125b**	cc	-0.077	0.241*	-0.287**	0.168	0.143	0.072
	p	0.460	**0.020**	**0.006**	0.111	0.180	0.515
**hsa-miR-217**	cc	-0.202	-0.148	-0.157	0.100	0.162	0.300**
	p	0.050	0.156	0.138	0.345	0.125	**0.007**
**CDKN2A**	cc	0.558***	0.070	0.173	-0.333**	-0.327**	-0.371**
	p	**0.0001**	0.563	0.160	**0.006**	**0.007**	**0.003**
**CIT (h)**	cc	-0.005	1.000	-0.007	0.055	0.025	0.215*
	p	0.960	.	0.947	0.605	0.812	**0.048**
**Cr t1/2**	cc	0.144	-0.007	1.000	-0.410**	-0.285**	-0.194
	p	0.173	0.947	.	**5.88E-05**	**0.006**	0.075
**MDRD4_3m**	cc	-0.606***	0.055	-0.410***	1.000	0.821***	0.702***
	p	**0.0001**	0.605	**5.88E-05**	.	**2.20E-23**	**6.81E-14**
**MDRD4_6m**	cc	-0.525***	0.025	-0.285**	0.821***	1.000	0.762***
	p	**0.0001**	0.812	**0.006**	**2.20E-23**	.	**2.65E-17**
**MDRD4_12m**	cc	-0.633***	0.215*	-0.194	0.702***	0.762***	1.000
	p	**0.0001**	**0.048**	0.075	**6.81E-14**	**2.65E-17**	.

* p<0.05

** p<0.01 and

***p<0.001.

Longer term function of renal allograft, measured as MDRD4 at 3, 6 and 12 months, revealed significant correlations with CDKN2A expression at all-time points (Table 2). When DGF-related microRNAs were analysed for their correlation with renal allograft function at post-operative day 3 (POD3), post-operative day 5 (POD5) and post-operative day 7 (POD7), the expression of hsa-miR-125b was positively correlated with MDRD4 at all time-points while hsa-miR-217 was associated with renal function on POD5 and POD7 (Table 3).

**Table 3:** Correlation matrix of miRNA expression and renal allograft function at post-operative days 3 (POD3), 5 (POD5) and 7 (POD7).

Spearman's rho		Post-operative day (POD) 3		Post-operative day (POD) 5		Post-operative day (POD) 5	
		Cr	MDRD4	Cr	MDRD4	Cr	MDRD4
**hsa-miR-125b**	cc	-0.230*	0.221*	-0.261*	0.238*	-0.315**	0.298**
	p	**0.027**	**0.035**	**0.012**	**0.022**	**0.002**	**0.004**
**hsa-miR-217**	cc	-0.199	0.185	-0.186	0.208*	-0.253*	0.256*
	p	0.057	0.078	0.076	**0.047**	**0.015**	**0.014**

### Multivariate analysis of factors related to Delayed Graft Function

Linear regression analysis of clinical parameters related to delayed graft function (cold ischaemia time, donor age, type of organ donation) and CDKN2A/p16 expression revealed significant associations with donor age and DCD status. Cold ischaemic time and CDKN2A/p16 expression were not significant in this model (Table 4 –model A).

**Table 4:** Regression analyses. A) Regression analysis of clinical parameters and CDKN2A only in the relation to Delayed Graft Function (DGF). B) Regression analysis of clinical and molecular parameters in multivariate model.

Variable (model A)	Est	Error	T value	P value
**Donor Age**	0.0095	0.0027	3.505	0.00083
**CDKN2A**	0.0075	0.0102	0.735	0.4650
**DCD donor**	0.3890	0.1110	3.573	0.0006
**Cold Ischaemic Time**	0.00016	0.0002	0.834	0.4073
**Variable (model B)**	**Est**	**Error**	**T value**	**P value**
**Donor Age**	0.00924	0.00238	3.874	0.0003
**CDKN2A**	0.00319	0.0090	0.354	0.7245
**DCD donor**	0.28216	0.1026	2.748	0.0078
**Cold Ischaemic Time**	0.0002	0.0002	0.855	0.3956
**hsa-miR-125b**	-0.39993	0.1040	-3.843	0.0002
**hsa-miR-217**	-0.222	0.0637	-3.489	0.0009

Further multivariate analysis performed with the inclusion of microRNAs, revealed that hsa-miR-217, hsa-miR-125b added additional power to the known clinical variables for delayed graft function in a biologically plausible context (Table 4 –model B).

### GRPSS model construction

Binary logistic regression was used with stepwise backwards conditional modelling to investigate the importance of all factors in determining the presence/absence of DGF post-transplant. This was applied to analyses for hsa-mir-217 and hsa-mir-125b expression levels relative to clinical parameters, to determine their predictive ability for individual performance criteria in the primary allograft cohort (n = 94). These included type of organ donation and previous transplant. These were added to the model and subtracted based on significance of input to the model. ECD criteria were not included as the specific formulation already includes donor age. CIT was excluded from the analysis as its occurrence correlates with hsa-miR-125b (Table 5).

**Table 5:** Binary logistic regression analysis of correlated microRNAs and clinical parameters with the respect to DGF—GRPSS model.

Variable	B	S.E.	Sig.	Odds ratio	95% C.I. for Odds ratio	
					Lower	Upper
**hsa-mir-217**	-1.987	0.787	0.012	0.137	0.029	0.641
**hsa-mir-125b**	-2.415	1.002	0.016	0.089	0.013	0.637
**Donor age**	0.069	0.021	0.001	1.072	1.028	1.118
**Type of organ donation**	-1.865	0.721	0.010	0.155	0.038	0.636

For these analyses DGF was defined as a need for dialysis within 7 days after transplantation, with the exception of post-operative hyperkalaemia.

Analysis of these microRNA expression levels, relative to the presence or absence of DGF using such modelling, indicated a capacity to predict pre-transplant the occurrence of DGF in 83% of cases, with an overall sensitivity of 61% and a specificity of 91% as determined by the prediction model. This model was tested in the context of type of organ donation, previous transplantation, CIT and donor gender (Table 5). Comparison of the model accuracy for correct prediction of DGF in relation to confounding factors is summarized in Table 6.

**Table 6:** Binary logistic regression analysis of correlated microRNAs and clinical parameters with the respect to DGF. Classification table for GRPSS model in relation to confounding factors.

**Predicted DGF**								
**Selected cases (S)**	All	Previous transplantation		Cold ischaemia time		Gender		
		No	Yes (S)	Above 12h (S)	Below 12h	Male (S)	Female	
**Observed DGF**								
**No**	91	83.3	90.7	92.6	75	85.3		87.5
**Yes**	61.5	80	71.4	73.7	57.1	83.3		62.5
**Overall percentage**	82.8	82.4	85.3	86.9	71	84.6		82.5

**Fig 3:**
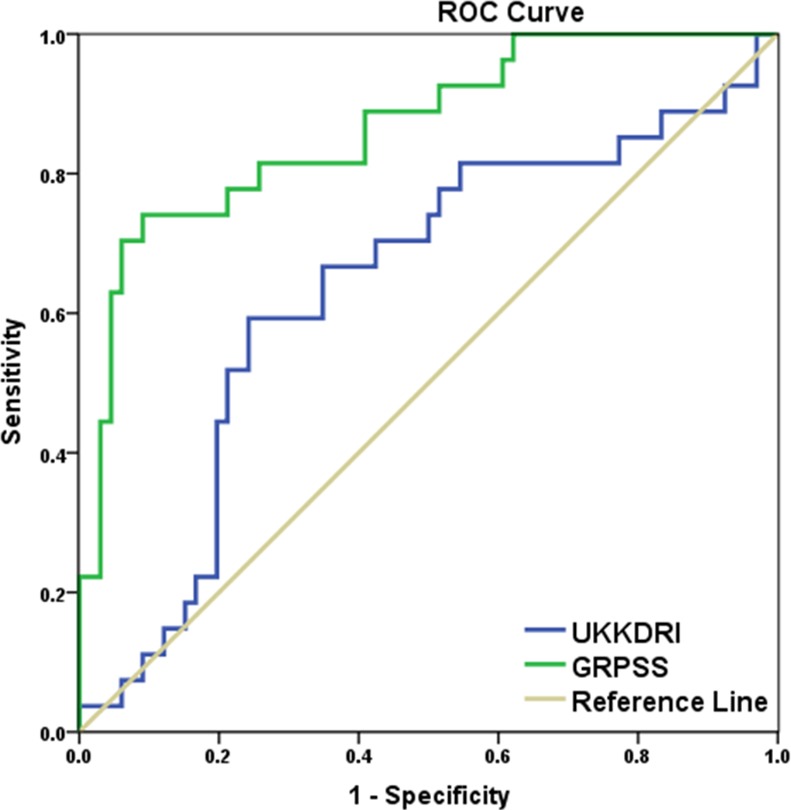
ROC analysis for GRPSS.

A direct comparison of the microRNA model, which we have termed the Glasgow Renal Performance Scoring System (GRPSS) with the most commonly used clinical method for determining allograft suitability in the UK, the UKKDRI [16] indicated that the GRPSS was better at predicting DGF occurrence (Table 7, Fig 3).

**Table 7:** ROC analysis comparing GRPSS model for DGF with UKKDRI.

Test Result Variable(s)	Area	Std. Error	Asymptotic Sig.	Asymptotic 95% Confidence Interval	
				Lower Bound	Upper Bound
**UKKDRI**	.636	.065	.041	.507	.764
**GRPSS**	.865	.043	.000	.781	.948

It should be noted that UKKDRI is a general performance scoring system and is not intended to solely detect the occurrence of DGF. The GRPSS is presented here as a method which is accurate and specific for DGF issues post-transplant.

These findings were then validated in an independent cohort (n = 24) using the same combination of microRNAs and donor age (S1 Data). This resulted in a model which successfully classified 70% of cases as DGF or non-DGF, with a sensitivity of 60% and a specificity of 77% however further analyses including type of organ donation and other confounding factors were not performed due to the small sample size.

## Discussion

We have clearly demonstrated that only miRNAs incorporated into a novel scoring system, the GRPSS, employed pre-transplant to assess renal allograft biopsies, can with high sensitivity and specificity predict the occurrence of DGF post-transplant. Consequently, we believe that the GRPSS has the potential to enhance current methodologies and advance the detection of poor performing allografts before they are transplanted, resulting in improved personalised care for transplant patients. In fact, this scoring system outperforms the UKKDRI scoring system for the detection of DGF, although the UKKDRI system was designed to improve organ allocation rather than detect specific post-transplant outcomes.

The molecular biology underpinning this modelling system is both consistent and biologically plausible. Both hsa-miR-217 and hsa-miR-125b, are associated with the regulation of the expression of the CDKN2 locus which has a proven regulatory role in biological ageing and renal function post-transplant [5,6,7]. Furthermore, these miRNAs have regulatory roles in cell-cell adhesion and related signalling pathways. These include TGF-β signalling, MAPK, PI3K-AKT, mTOR cascades, actin skeleton remodelling and endocytosis [17]. Notably, they target pathways related to cell stress and survival in model organisms [18] and thus provide a biological signature consistent with data derived from different taxa, including dipteran insects, nematode worms, yeast as well as mammals. The TGF-β cascade has previously been linked to renal function, both in a reno-protective capacity and in renal inflammation and fibrosis [19].

In particular, hsa-miR-125b and hsa-miR-217 are involved in the regulation of a broad range of cellular/biological processes that intersect on the MAPK signalling and the PI3K-AKT pathways. Critically, these link regulations of immune responses via modulation of T and B cell receptor and chemokine signalling, as well as the remodelling of the extracellular matrix, including regulation of actin cytoskeleton, adherence junction and focal adhesions [17], all of which have proven roles in allograft performance. Notably, the MAPK signalling pathway has been linked to both chronic and acute kidney disease, where it has a dual function, mediating kidney injury responses as well as having a role in renal stress resistance [20].

Previous studies have also demonstrated that increased expression of hsa-miR-217 is associated with suppression of Ras/MAPK and PI3K-AKT [21,22], indicating that these pathways may be up-regulated by the suppressed expression of this miRNA.

Interestingly, the MAPK and TGF-β cascades, play important roles in the regulation of microRNA biogenesis and can induce specific phenotypes depending on cellular context [23]. It should also be noted that the TGF-β pathway is involved in the activation of a broad range of MAPK pathway components including RAS/MEK/ERK1/2 or Rho/JNK, independently of SMAD proteins, but still affecting their phosphorylation status and thus modulating their activity as transcription factors.

hsa-miR-125b has been linked to the promotion of a pro-inflammatory state by the modulation of numerous target genes including matrix metalloproteinase, growth factors and BCl2 family members. This miRNA is also critical for immunological host-defence responses and autoimmunity, as well as immune cell differentiation and their responses to the external stimuli i.e., IL-4 and INF-γ [24]. Our data indicate a role for such processes in determining the long-term success of allograft transplantation. These data are also in keeping with previous reports [25,26] and treatment regimes targeting leukocyte adhesion molecules, which have been shown to be critical in a range of kidney disorders including allograft rejection [27,28].

Our data do not readily agree with recent studies which have focused solely on miRNA analysis in post-transplant renal allografts (12–26 months post-transplantation) [17]. These studies describe miRNA profiles in kidneys that have already undergone acute rejection (AR) episodes, which thus serve as a ‘finger print’ of extant rejection. Interestingly, some of these microRNAs showed differential expression in pre-transplant biopsies, including hsa-miR-125b, indicating a potentially ongoing role for these miRNAs in the development and progression of conditions leading to AR.

One hypothesis in keeping with our observations, is that organs displaying more biological age and consequently poorer post-transplant function [29] will have a higher risk of rejection, due to ischemic, metabolic and physical trauma incurred during the transplant process, resulting in leakage of cellular chromatin and mitochondrial proteins from damaged cells in such organs, which might trigger immune responses in the recipient. This hypothesis is in keeping with the correlations we have observed between pre-transplant levels of miRNAs involved in the regulation of cellular bio-ageing. The more senescent/damaged cells present within the organ, the more intra-cellular material that might leak into the recipient circulation. Consequently, the likelihood of poor post-transplant performance is dependent on the level of senescence related pre-implantation damage in the organ; other factors may include tissue remodelling, accumulation of ECM components and consequent promotion of a pro-inflammatory environment by senescent cells. Our data suggest a mechanism which involves organ condition at point of acquisition combined with rapid response damage, via miRNA levels, resulting in an acceleration of the senescence associated phenotype within the donor organ. This accelerated bio-ageing which occurs between organ retrieval and transplantation ultimately determines post-transplant outcomes. In this instance, we have demonstrated that the levels of two such rapid response elements (hsa-miR-217 and hsa-miR-125b) are significantly altered in organs which experience DGF post-transplantation

Our data provides clear evidence that pre-transplant analysis of miRNA related to cellular bio-ageing, cell stress and damage responses is useful in determining post-transplant function of renal allografts. We believe this work demonstrates the considerable potential of miRNAs for assaying the status of donor organs, and to improve the assessment of marginal organs by providing more accurate pre-transplant prediction of post-transplant function. Notably, the turnaround time for the assays involved in implementing this molecular analysis is only 4 hours, which is within the time window for organ cross matching, indicating the ease of clinical translation of this approach. We therefore believe our findings merit larger scale studies to (a) develop a robust predictive tool for donor organs, and (b) validate that tool in different populations.

## Supporting Information

S1 Data Supplementary Materials and Methods for Validation Cohort. The materials and methods for the validation cohort from Innsbruck and Tubingen, includes details on sample collection, RNA isolation and microRNA expression profiling. **Table A** contains the cohort characteristics for the validation cohort.
(DOCX)
