# Correction: Identification of Molecular Markers of Delayed Graft Function Based on the Regulation of Biological Ageing

**DOI:** 10.1371/journal.pone.0151506

**Published:** 2016-03-10

**Authors:** Dagmara McGuinness, Johannes Leierer, Olivier Shapter, Suhaib Mohammed, Marc Gingell-Littlejohn, David B. Kingsmore, Ann-Margaret Little, Julia Kerschbaum, Stefan Schneeberger, Manuel Maglione, Silvio Nadalin, Sylvia Wagner, Alfred Königsrainer, Emma Aitken, Henry Whalen, Marc Clancy, Alex McConnachie, Christian Koppelstaetter, Karen S. Stevenson, Paul G. Shiels

There is an error in affiliation for authors Johannes Leierer, Julia Kerschbaum and Christian Koppelstaetter. The correct affiliation is: Department of Internal Medicine IV (Nephrology and Hypertension), A-6020 Medical University Innsbruck, Austria.
