# Supplementary material for: Identification of Molecular Markers of Delayed Graft Function Based on the Regulation of Biological Ageing
Source: PLoS One. 2016 Jan 6;11(1):e0146378. doi: 10.1371/journal.pone.0146378 (PMC4703336; doi:10.1371/journal.pone.0146378)
Supplement: S1 Data — The materials and methods for the validation cohort from Innsbruck and Tubingen, includes details on sample collection, RNA isolation and microRNA expression profiling. Table A contains the cohort characteristics for the validation cohort. (DOCX) [file pone.0146378.s001.docx]

**S1 Supplementary data**

**Table A**. Innsbruck and Tübingen cohort characteristics*

| **Variable** | **Mean / proportion** | **Standard deviation**  **(if applicable)** |
| --- | --- | --- |
| **Recipient gender (males/females)** | 14/10 |  |
| **Donor gender (males/females)** | 15/9 |  |
| **Recipient age (years)** | 57.5 | 12.0 |
| **Donor age (years)** | 57.3 | 17.9 |
| **Donor serum creatinine (µmol/L)** | 76.3 | 27.0 |
| **Serum creatinine level at 6 months (µmol/L)** | 157.7 | 66.3 |
| **MDRD4 at 6 months (ml/min/1.73m^2^)** | 45.8 | 21.5 |
| **Serum creatinine level at 1 year (µmol/L)** | 155.5 | 66.4 |
| **MDRD4 at 12 months (ml/min/1.73m^2^)** | 47.5 | 24.1 |
| **Cold ischaemic time (hours)** | 13.3 | 5.2 |
| **DGF** | 10/24 |  |

***Note:** The total 34 samples were analysed for this cohort, however some clinical data were not available. Samples with missing clinical data were automatically listwise excluded from further analyses.

**Supplementary Materials and Methods**

**MicroRNA expression in renal biopsies and data analysis (Innsbruck and Tübingen)**

**RNA Extraction and Quality control**

Zero-hour kidney biopsies were removed from RNA®*later*(Ambion, USA), fragmented and homogenized. Total RNA was isolated according to the manufacturer’s protocol for the RNeasy Mini Kit (Qiagen, Valencia, CA). RNA yield was determined spectrophotometrically using a Nanodrop 1000 (ThermoFisher Scientific, Wilmington, USA). The validation cohort had a separate ethical approval granted (Innsbruck Medical University and Universitätsklinikum Tübingen) and full written consent was collected for each subject.

**miRNA expression profiling.**

A validated miRNA screening pipeline was used, which allowed accurate and sensitive expression analysis of 755 miRNAs by means of real-time quantitative PCR with hydrolysis probe-based miRNA assays (Mestdagh et al., 2008). Shortly, total RNA was reverse transcribed using the Megaplex RT stem-loop primer pool A and B (Applied Biosystems, Life Technologies Corporation, Carlsbad, CA, USA). The cDNA was then pre-amplified by means of a 12-cycle PCR reaction with a miRNA specific forward primer and universal reverse primer. Finally, a dilution of pre-amplified miRNA cDNA was used as input for a 40-cycle qPCR reaction with miRNA specific hydrolysis probes and primers (Applied Biosystems, Life Technologies Corporation, Carlsbad, CA, USA). All reactions were performed on Applied Biosystems 7900 HT using the gene maximization strategy. Three endogenous small RNA control targets (RNU44, RNU48 and U6) were included to check for intra-run and inter-run variation. The Cq values were subsequently analysed in qbasePLUS, Cq values above 32 were considered noise and excluded [1]. miRNA expression data were normalized using the modified global mean in qbasePLUS [2].

Each of the qPCR amplification was performed in triplicates and the gathered data were normalized with qBasePlus (Biogazelle, Zwijnaarde, Belgium) against the housekeeping genes G6pdx, Tbp, and Tuba1a.

**References**

1. Hellemans J, Mortier G, De Paepe A, Speleman F, Vandesompele J (2007) qBase relative quantification framework and software for management and automated analysis of real-time quantitative PCR data. Genome Biol 8: R19.

2. D'Haene B, Mestdagh P, Hellemans J, Vandesompele J (2012) miRNA expression profiling: from reference genes to global mean normalization. Methods Mol Biol 822: 261-272.
